# Analysis of dietary fats intake and lipid profile in Chilean patients with glucose transport type 1 deficiency syndrome: similarities and differences with the reviewed literature

**DOI:** 10.3389/fnut.2024.1390799

**Published:** 2024-05-16

**Authors:** María Florencia Salazar, María Jesús Leal-Witt, Valentina Parga, Carolina Arias, Verónica Cornejo

**Affiliations:** ^1^Laboratorio de Genética y Enfermedades Metabólicas del Instituto de Nutrición y Tecnología de los Alimentos (INTA), Universidad de Chile, Santiago, Chile; ^2^Instituto Nacional de Deporte, Santiago, Chile; ^3^Hospital Clínico UC Christus, Santiago, Chile

**Keywords:** transporter type 1 deficiency syndrome, ketogenic diet, fatty acid intake, lipids profile, dyslipidemia, transaminases

## Abstract

**Introduction:**

Glucose transporter type 1 deficiency syndrome (GLUT1-DS) is a neurological disorder caused by mutations in the SLC2A1 gene. The main treatment is ketogenic diet therapy (KDT), which changes the brain’s energy substrate from glucose to ketone bodies. The diet controls seizures, but there may be side effects such as dyslipidemia. This study aimed to describe the type of fats ingested by the Chilean cohort of patients with GLUT1-DS and analyze for alterations in the lipid profile.

**Methods:**

A GLUT1-DS group and a control group were formed, each with 13 subjects who were matched by age, gender, and nutritional status. Anthropometry, dietary intake, including types of fat, and blood tests were evaluated (lipid and liver profile, and 25-hydroxyvitamin D levels).

**Results:**

A high-fat diet, especially saturated fat, was identified in the GLUT1-DS group (38% of total calories), with the use of medium-chain triglycerides (17% of total calories). In addition, GLUT1-DS participants had a higher intake of monounsaturated (MUFA) and polyunsaturated (PUFA) fats and adequate consumption of omega-3 (2% of total calories). Despite the GLUT1-DS group receiving on average 80% of its total energy as fats, it is important to highlight that 50% are MUFA+PUFA fats, there were no significant differences in the lipid and liver profile compared to the control group.

**Conclusion:**

KDT did not negatively impact lipid profile, despite a high intake of fats. It is important to monitor lipid profiles, in a personalized and constant manner, to prevent future nutritional risks.

## 1 Introduction

Glucose transporter type 1 deficiency syndrome (GLUT1-DS) (OMIM 606777) is a defect in the SLC2A1 gene, which encodes the type 1 glucose transporter and is located on chromosome 1p34.2 (1). This transporter is expressed mainly in erythrocytes and the blood-brain barrier. Most cases of GLUT1-DS are inherited in an autosomal dominant manner, presenting heterozygous de *novo* mutations. Worldwide, a prevalence of approximately 105,000 cases with GLUT1-DS has been estimated, with an expected incidence of 1.65 to 2.2 per 100,000 live births (2). This syndrome is a neurological disorder that affects brain energy metabolism and has a great phenotypic variability. The classic presentation debuts with early epileptic encephalopathy and is associated with developmental delay, acquired microcephaly, lack of motor coordination, and spasticity (3).

Seizures typically manifest within the first 4 months of life, presenting with episodes of apnea, cyanotic spasms, atony attacks, and distinctive eye movements. Their occurrence is notably higher in cases involving atonic seizure-myoclonus or early-onset absences (3). Suspected cases of GLUT1 deficiency syndrome (GLUT1-DS) often present with hypoglycorrhachia (cerebrospinal fluid glucose <40 mg/dL), alongside normal serum glucose levels and a cerebrospinal fluid (CSF) glucose to blood glucose ratio below 0.55 (reference > 0.60). Differential diagnosis must exclude central nervous system (CNS) infections, while CSF lactate levels may vary. Confirmatory diagnosis necessitates molecular analysis for variants in the SLC2A1 gene. However, it’s crucial to note that current guidelines acknowledge the possibility of GLUT1-DS even without detectable variants in SLC2A1 (2).

GLUT1-DS is considered a treatable energetic defect, and the standard recommended therapy is the ketogenic diet (KDT). It consists of changing the fuel source, mainly for the brain, from using glucose to ketone bodies as energy (2). The expert committee for GLUT1-DS recommends using KDT with a 3:1 ratio, which means high fat intake (70–90% of total energy), low carbohydrate intake (3–15% of total energy), and protein (according to needs by age), thus achieving ketogenesis with levels of β-hydroxybutyrate acid (β-OHB) between 1.5 to 5 mmol/L (4, 5). Dietary management has proven to be effective in controlling seizures. Approximately 80% of patients have achieved a reduction in more than 90% of seizures, and 64% do not take anticonvulsant drugs. Furthermore, improvement in abnormal movements and cognitive development has been observed; however, language ataxia persists despite KDT (2).

Long-term treatment can cause adverse effects, such as slow weight gain, osteopenia, and dyslipidemia. Regarding dyslipidemia, between 14% and 59% of patients with classic KDT have presented an increase in triglyceride and low-density lipoprotein (LDL) levels, and 60% have developed hypercholesterolemia. Furthermore, adverse factors occur more frequently when starting the diet and then tend to normalize (4). Therefore, close monitoring and follow-up are recommended to prevent long-term adverse effects (6, 7).

This work aimed to describe the type of fats ingested by the Chilean cohort of patients with GLUT1-DS in KDT and determine if there is a relation with lipid profile.

## 2 Materials and methods

### 2.1 Study design

We conducted a cross-sectional observational study.

### 2.2 Participants

*Inclusion criteria*: Subjects with GLUT1-DS, who had been with KDT for 1 year or more, with a molecular study of the SCL2A1 gene, in follow-up at the Metabolic Diseases clinic at the Instituto de Nutrición y Tecnología de los Alimentos (INTA) of University of Chile, with follow-up records, ketonemia and signed informed consent.

*Exclusion criteria*: Subjects who had infectious, inflammatory, traumatic, and neoplastic conditions in the last month before entering this study. Subjects from the GLUT1-DS group who had suspended the KDT for at least 1 month and/or without control of ketonemia. Control subjects, with some associated pathology, on some type of special diet or nutritional treatment different from a common dietary pattern for their age.

The GLUT1-DS group had 13 subjects who met the inclusion criteria. A control group was considered, including 13 healthy subjects with normal diets and matched by gender, age, and nutritional status with the GLUT1-DS group. Anthropometric evaluation: Weight and height were evaluated using a digital scale and precision stadiometer (0.1 kg and 0.1 cm, respectively), in the Frankfurt position, with light clothing with no shoes. We calculated weight/height (W/H), body mass index/age (BMI/A) and height/age (H/A) z-scores. For nutritional diagnosis in those under 18 years of age, the child growth patterns of the World Health Organization (WHO) 2007 were used according to sex and age (8).

### 2.3 Ketogenic diet (KDT)

The ketogenic diet therapy (KDT) considered the following distribution of macronutrients: 3–15% of total energy from carbohydrates, 70–90% from lipids, and proteins adjusted based on age and gender recommendations (9).

Special ketogenic formula: In the GLUT1-DS group, 12 subjects received a special ketogenic formula with a 4:1 ratio (Ketovolve^®^), subsidized by the Government of Chile. Only one adult case did not consume this product or similar.

Supplementation: All GLUT1-DS subjects who needed to meet vitamin and mineral requirements were pharmacologically supplemented.

### 2.4 Analysis of dietary intake

Twenty-four-hour dietary recall questionnaires were applied for 3 days (2 weekdays and 1 weekend day). Intake of energy (kcal/day), protein (% of total energy; %E), carbohydrates (%E), total fat (%E), cholesterol (mg/day), vitamin C (mg/day), and vitamin D (IU/day) were measured. In addition, the analysis of saturated fat (SFA, %E), monounsaturated fat (MUFA; %E), and polyunsaturated fat (PUFA; %E) was included. Also, the breakdown of omega 3 fatty acid family as total omega 3 (%E), α-linolenic acid (ALA; %E), eicosapentaenoic acid (EPA; %E), and docosahexaenoic acid (DHA; %E) was done. The INTA food composition table from 2011 and 2018 was used to obtain this information (10, 11). The amounts of nutrients recorded were compared with the recommended daily intake (dietary reference intake; RDI) according to gender and age, considering between 90% and 110% of the RDI as normal (Supplementary Table 1) (12–14).

### 2.5 Biochemical parameter measurement

GLUT1-DS Group: The level of ketones or β-hydroxybutyrate (β-OHB) was measured in capillary blood with a minimum fast of 8 h through a hemoglucotest. Ketogenesis was considered when the β-OHB value was above 1.5 mmol/L and not higher than 5 mmol/L. Glycaemia was evaluated, which was measured consecutively with ketonemia and was considered an acceptable value between 65 to 95 mg/dL.

Control group: Ketonemia under 0.6 mmol/L and glycemia between 90 to 100 mg/L.

Together, a 5 ml sample of venous blood was extracted from both groups in heparinized vials to determine plasma levels of aspartate aminotransferase (AST; ref <52 U/L), alanine transaminase (ALT; ref ≤ 39 U/L), gamma-glutamyl transferase (GGT; ref ≤ 23 U/L), lactate dehydrogenase (LDH; ref 135 - 345 U/L), alkaline phosphatase (ALP; ref 35 - 269 U/L), international rationalized index (INR; ref ≤ 1), total cholesterol (TC; ref <200 mg/dL), high-density lipoprotein (HDL; ref > 40 mg/dL), low-density lipoprotein (LDL; ref <100 mg/dL); very low-density lipoprotein (VLDL; ref <30 mg/dL), triglycerides (TG; ref <150 mg/dL) and vitamin D (25-OHD; ref: normal ≥ 30 ng/mL, insufficiency ≤ 29 and deficiency ≤ 20 ng/mL).

### 2.6 Statistics

A descriptive analysis was performed on subjects with GLUT1-DS and matched controls. For each variable, the distribution was checked through the Shapiro–Wilk test and the mean ± standard deviation (SD) or the median with the interquartile range (IQR 25–75) was considered in the analysis. The difference between groups (GLUT1-DS and control group) was evaluated with the Mann–Whitney test for two groups, for variables with a non-normal distribution, and with the Student *t*-test for variables with a normal distribution. Linear associations for all variables, due to the number of subjects, were performed using the Spearman test. A *p* < 0.05 was considered statistically significant. JMP 17.1 statistical software was used to perform the analysis.

### 2.7 Ethical considerations

The study was evaluated and approved by the Ethics Committee of INTA, Universidad de Chile, on April 14, 2021. To complete all the required analyses, parents and/or caregivers signed an informed consent form and, in those older than 8 years of age, an assent signed by the subjects themselves.

## 3 Results

Table 1 reports the characteristics of the two groups, each with 13 subjects, 7 men and 6 women. There was one adult, and the rest were under 18 years of age, with an average age of 10.2 ± 5.5 years (GLUT1-DS Group) and 9.9 ± 5.4 years (Control Group). GLUT1-DS Group patients have been on this treatment for an average of 59 months (minimum 16 months and maximum 251 months). For nutritional status, in each group, there was 1 subject at risk of malnutrition, 6 normal weight, 3 overweight, and 3 obese persons.

**TABLE 1:** Clinical characteristics among subjects in the GLUT1-DS and control groups.

	GLUT1-DS Group (*n* = 13)		Control Group (*n* = 13)		
	Mean ± SD	Median (IQR)	Mean ± SD	Median (IQR)	*p*-value^†^
Age (years)	10.2 ± 5.5	8.7 (6.7; 13.9)	9.9 ± 5.4	9.4 (6.0; 12.8)	NS
*Weight (kg)	33.9 ± 17.0	25.5 (23.0; 43,5)	38.5 ± 20.8	29.0 (26.0; 49.8)	NS
BMI for age or Weight for age z-score	0.5 ± 1.2	0.6 (−0.3; 1.5)	0.7 ± 1.1	0.6 (−0.2; 1.4)	NS
Height (cm)	130.0 ± 0.2	124.0 (111.0; 151.0)	135.9 ± 0.2	133.0 (127.0; 149.0)	NS
Height for age z-score	−1.1 ± 0.9	−1.0 (−1.8; −0.4)	0.4 ± 1.0	0.3 (−0.4; 0.9)	0.001

Values are expressed as mean ± standard deviation and median (interquartile range), as appropriate. The distribution variable was considered for data analysis, we indicate with asterisks (*) the non-normal distribution varible.

^†^Significant difference (*p* < 0.05) by the Mann–Whitney test or Student *t*-test as appropriate. GLUT1-DS, Glucose Transporter Type 1 Deficiency Syndrome; BMI, body mass index.

No difference was observed between groups in age, weight, height, weight for height, and BMI for age z-score. However, a significant difference was observed in height for age z-score (*p* = 0.001), with a lower z-score observed for the GLUT1-DS group compared to the control group but within normal ranges (Table 1).

No significant difference was observed in caloric intake, but there was a difference between groups regarding the distribution of the caloric molecule of macronutrients (carbohydrate, protein and fat). In the GLUT1-DS group, when on KDT, 81% of the total caloric molecule came from fat, 13% from proteins, and 4% from carbohydrates. While the control group had a normal distribution with 54% carbohydrates, 27% fats, and 19% proteins (Table 2).

**TABLE 2:** Comparison of nutrient intake between the GLUT1-DS and control groups.

	GLUT1-DS group (*n* = 13)		Control group (*n* = 13)		
	Mean ± SD	Median (IQR)	Mean ± SD	Median (IQR)	*p*-value^†^
Energy (Kcal/day)	1614.2 ± 428.6	1551.3 (1355; 1912.0)	1401.1 ± 253.2	1423 (1203; 1512)	NS
Ketogenic ratio	2:1 ± 0.5	2:1 (1.7:1; 2.5:1)	NA		
Protein (%E)	13.2 ± 3.9	12.7 (10.8; 14.5)	18.6 ± 1.6	18.5 (17.6; 19.7)	0.002
*Carbohydrates (%E)	5.5 ± 3.5	3.9 (2.5; 8.3)	53.3 ± 6.7	54.0 (49.3; 58.9)	<0.001
*Fat (%E)	80.0 ± 5.9	81.2 (77.2; 83.9)	27.1 ± 6.7	26.8 (20.3; 33.4)	<0.001
*Cholesterol (mg/day)	245.3 ± 201.2	228.0 (37.9; 421.7)	178.3 ± 160.5	107.8 (99.3; 170.9)	NS
*Vitamin C (mg/day)	460.4 ± 240.1	581.0 (179.0; 618.0)	60.9 ± 32.2	71 (37.0; 80.0)	<0.001
*Vitamin D (IU/day)	889.1 ± 598.8	708.0 (556; 855)	81.0 ± 99.7	64 (21.3; 82.7)	<0.001

Values expressed as mean ± standard deviation and median (interquartile range), as appropriate. The distribution variable was considered for data analysis, we indicate with asterisks (*) the non-normal distribution variable.

^†^Significant difference (*p* < 0.05) by the Mann–Whitney test* or Student *t*-test as appropriate. GLUT1-DS, Glucose Transporter Type 1 Deficiency Syndrome; %E, percentage of total energy; ALA, α-linolenic acid; EPA, eicosapentaenoic acid; DHA, docosahexaenoic acid; NA, not applicable; NS, Non-significative.

It was observed that the GLUT1-DS group had a higher intake of saturated fat (69.4 g/day, 38 %E), MUFA 40.4 g/day (20 %E) and PUFA 31.3 g/day (17%E) compared to the control group which had 16.7 g/day (11%E), 14.1 g/day (8%E) and 8.2 g/day (5%E), respectively (Table 3). The control group reported a consumption of saturated fat above the recommended amounts (Supplementary Table 1).

**TABLE 3:** Comparison of different types of fat intake between the GLUT1-DS and control groups.

	GLUT1-DS group (*n* = 13)		Control group (*n* = 13)		
	Mean ± SD	Median (IQR)	Mean ± SD	Median (IQR)	*p*-value^†^
*Fat (%E)	80.0 ± 5.9	81.2 (77.2; 83.9)	27.1 ± 6.7	26.8 (20.3; 33.4)	<0.001
*SFA (%E)	38.7 ± 8.9	38.4 (34.8; 41.3)	10.5 ± 2.5	10.8 (8.1; 12.2)	<0.001
MUFA(%E)	22.4 ± 5.5	19.8 (18.3; 26.6)	8.8 ± 3.2	7.5 (6.9; 11.5)	<0.001
*PUFA (%E)	17.7 ± 5.6	16.9 (13.9; 20.5)	5.3 ± 2.5	4.9 (3.5; 5.7)	<0.001
*Omega 3 (mg/day)	3149.3 ± 1942.2	3441.3 (1174.0; 5228.0)	51.3 ± 88.3	0.0 (0; 66.7)	<0.001
Omega 3 (%E)	1.8 ± 1.0	1.9 (1.0; 2.3)	0.03 ± 0.06	0.0 (0; 0.03)	<0.001
*ALA (mg/day)	2576.9 ± 1741.6	2359.0 (1174; 3351)	51.3 ± 88.3	0.0 (0; 66.7)	<0.001
*EPA + DHA (mg/día)	572.4 ± 880.7	315.0 (0; 546)	ND	ND	<0.003
*Omega 6 (mg/day)	14417.9 ± 9480.6	14238 (5728; 19427)	ND	ND	<0.001
*Omega 6 (%E)	8.3 ± 5.6	8.1 (3.8; 10.3)	ND	ND	<0.001

Values are expressed as mean ± standard deviation and median (interquartile range), as appropriate. The distribution variable was considered for data analysis, we indicate with asterisks (*) the non-normal distribution variable.

^†^Significant difference (*p* < 0.05) by the Mann–Whitney test* or Student *t*-test as appropriate. GLUT1-DS, Glucose Transporter Type 1 Deficiency Syndrome; %E, percentage of total energy; SFA, saturated fat; MUFA, monounsaturated fat, PUFA, polyunsaturated fat; ALA, α-linolenic acid; EPA, eicosapentaenoic acid; DHA, docosahexaenoic acid; ND, not determined.

The GLUT1-DS group, as part of the KDT, consumed medium chain triglycerides (MCT), equivalent to 45% of the total saturated fat intake, representing 17% of total energy consumed. Considering the form of MCT delivery, 70% was ingested as a commercial oil and 30% from the special ketogenic formula.

The GLUT1-DS group had a higher consumption of MUFA fats (22.4 ± 5.5 %E) (Table 3) compared to the control group (8.8 ± 3.2 %E), with the main food sources being avocado, nuts, olive, and the special ketogenic formula.

Regarding the consumption of omega 3, including ALA, EPA, and DHA, it was observed that 11 of the 13 subjects with GLUT1-DS managed to meet their requirements. The main food sources were: canola oil, vegetable oil, and fish oil. The control group had a significantly lower intake of these fatty acids, failing to meet the recommendations established for a healthy population.

It is of metabolic importance to maintain an optimal ratio of 5:1 between the family of omega 6 and omega 3 fatty acids for adequate synthesis of these essential fatty acids at the peroxisomal level (12, 15). This ratio could only be determined in the GLUT1-DS group, which had an average intake of 14.4 g/day of omega 6 and 3.2 g/day of omega 3, resulting in a ratio of 4.5:1.

It was observed that the GLUT1-DS group had an average intake of vitamin C of 460.4 ± 240.1 mg/day and 889.1 ± 598.8 IU/day of vitamin D, higher intakes than those detected in the control group (*p* < 0.001) (Table 2). We note that for the GLUT1-DS group, either vitamin C and Vitamin D, 95% and 100% respectively came from ketogenic formula and pharmacological supplementation. However, four GLUT1-DS subjects did not meet the recommendations for vitamin D and one for vitamin C. In the control group, 4 subjects did not meet the vitamin C recommendation, and none met the vitamin D intake recommendation.

When comparing levels of 25-OHD, the GLUT1-DS group had levels 40.3 ± 13.3 ng/ml compared to 26.0 ± 8.4 ng/ml in the control group (*p* < 0.0012) (Table 4). Within the control group, six cases were classified as having insufficient vitamin D levels and three cases were classified as having deficiency. In the GLUT1-DS group, there were two cases with insufficient levels.

**TABLE 4:** Liver profile and vitamin D levels for the GLUT1-DS and control groups.

	GLUT1-DS group (*n* = 13)		Control group (*n* = 13)		
	Mean ± SD	Median (IQR)	Mean ± SD	Median (IQR)	*p*-value^†^
Glycemia (mg/dL)	77.3 ± 10.3	77 (71; 84)	78,5 ± 4.6	77 (77; 79)	NS
Lactate dehydrogenase (U/L)	197.6 ± 36.8	190 (181; 211)	226.7 ± 42.6	228 (208; 254)	NS
* Alkaline phosphatase (U/L)	247.5 ± 182.1	191 (173; 242)	243.2 ± 99.3	254 (183; 305)	NS
*AST (U/L)	39.0 ± 47.5	25 (15.5; 42.5)	26.0 ± 7.7	25 (22; 32)	NS
*ALT (U/L)	64.7 ± 99.0	23 (14.25; 44.3)	17.4 ± 9.0	15 (12; 21)	NS
*GGT (U/L)	14.8 ± 5.0	13.5 (11; 18)	12.2 ± 5.0	11 (10; 12)	NS
INR	1.1 ± 0.1	1.06 (1.0; 1.2)	1.0 ± 0.1	1.04 (1.02; 1.01)	NS
*Vitamin D (ng/mL)	40.3 ± 13.3	33.9 (30.4; 50.8)	26,0 ± 8.4	24.4 (21.3; 30.1)	0.0012

Values expressed as mean ± standard deviation and median (interquartile range), as appropriate. The distribution variable was considered for data analysis, we indicate with asterisks (*) the non-normal distribution variable.

^†^Significant difference (*p* < 0.05) by the Mann–Whitney test* or Student *t*-test as appropriate. GLUT1-DS, Glucose Transporter type 1 Deficiency Syndrome; ALT, Alanine Amino Transferase; AST, Aspartate Amino Transferase; GGT, γ-glutamyl transferase; INR, international normalized index; NS, non-significative.

When comparing ketonemia and glycemia levels between the control and GLUT1-DS groups, significant differences were found. The GLUT1-DS group on KDT had on average a fasting ketonemia value of 2.8 ± 2.0 mmol/L (median 2.3, IQR 1.2; 4.2) compared to 0.3 ± 0.2 mmol/L (median 0.3, IQR 0.2; 0.3) in the control group (*p* < 0.002). Regarding glycemia, the GLUT1-DS group had an average value of 75.2 ± 10.8 mg/dL (median 76, IQR 68- 78) and the control group of 83.0 ± 7.0 mg/dL (median 83, IQR 78- 86) (*p* < 0.042).

No differences were observed between groups (Tables 4, 5) for the rest of the biochemical parameters evaluated. In the GLUT1-DS group, three adolescent subjects had ALP levels above the reference value, another case had high levels of AST and ALT enzymes, and two other GLUT1-DS subjects had ALT above the reference range. In the control group, no transaminase alterations were observed, but five subjects in the pubertal stage presented elevation of the ALP enzyme.

**TABLE 5:** Lipid profile for the GLUT1-DS and control groups.

	GLUT1-DS group (*n* = 13)		Control group (*n* = 13)		
	Mean ± SD	Median (IQR)	Mean ± SD	Median (IQR)	*p*-value^†^
TC (mg/dL)	154.3 ± 27.6	148 (138; 169)	146.2 ± 17.8	142 (138; 167)	NS
HDL (mg/dL)	60.7 ± 14.0	63.6 (55.7; 69.3)	54.2 ± 12.6	52.5 (46.9; 59.2)	NS
LDL (mg/dL)	80.8 ± 24	79 (62; 92)	75.2 ± 17.6	73.8 (61.8; 86.9)	NS
*VLDL (mg/dL)	12.6 ± 6.3	11 (9.2; 12.3)	17.0 ± 8.7	15.3 (11.3; 19.3)	NS
*TG (mg/dL)	63.4 ± 31.6	55 (46; 62)	85.0 ± 43.6	77 (56; 97)	NS
*TC/HDL	2.7 ± 0.9	2.6 (2; 3)	2.9 ± 0.7	3 (2; 3)	NS
*TC/TG	2.7 ± 0.7	3 (2; 3)	2.0 ± 1.0	2 (1; 3)	NS

Values expressed as mean ± standard deviation and median (interquartile range), as appropriate. The distribution variable was considered for data analysis, we indicate with asterisks (*) the non-normal distribution variable.

^†^Significant difference (*p* < 0.05) by the Mann–Whitney test* or Student *t*-test as appropriate. GLUT1-DS, Glucose Transporter type 1 Deficiency Syndrome; TC, Total Cholesterol; HDL, High Density Lipoprotein; LDL, Low Density Lipoprotein; VLDL, Very Low Density Lipoprotein; TG, Triglycerides; NS, non-significative.

In the GLUT1-DS group, there was one patient with slightly high cholesterol (208 mg/dL), and another patient with high levels of triglycerides (160 mg/dL) and LDL (125 mg/dL), however, both subjects were in the normal nutritional status category.

In the control group, two subjects were found to have an altered lipid profile. One of them with obesity and high levels of VLDL (40 mg/dL) and triglycerides (199 mg/dL), and HDL below recommended levels (34 mg/dL). The other subject was overweight and had an HDL below recommended levels (35 mg/dL).

A positive correlation was observed between saturated fat consumption and ALT concentration in blood (ρ = 0.47; *p* = 0.017). An inverse correlation was observed between MUFA fat intake, triglycerides (ρ = −0.527; *p* = 0.005), and VLDL (ρ = −0.53; *p* = 0.005).

## 4 Discussion

A ketogenic diet is the first-line treatment for patients with GLUT1-DS, which should be started as soon as possible to reduce seizures and involuntary movements caused by a lack of energy in the CNS (4).

When comparing our observed findings with a study that used a ketogenic diet in patients with GLUT1-DS, and which evaluates the distribution of different types of fats, we can see that our GLUT1 cohort had a higher intake of proteins (13%E versus 8%E), saturated fats (38%E versus 31%E), PUFA (17%E versus 12%E), and a lower intake of total fat (81%E versus 87%E) and cholesterol (228 mg versus 277 mg) (16). This study reports the exclusive use of a classic ketogenic diet but does not provide details on the type of fats used, such as EPA and DHA (16). The differences found mainly in the contribution of saturated fats could be due to the fact that our cohort consumed 17% of the total fats as MCT, which could be influencing a greater intake of saturated fats. However, with a higher consumption of MCT, the total fat intake was reduced, and ketonemia was maintained within the recommended ranges for KDT.

There is evidence supporting the association between a high consumption of saturated fat and an increased risk of cardiovascular disease, as it contributes to increased total and LDL cholesterol (17). When MCT oil is used as a complement to a classic ketogenic diet, it aims to increase ketone production and prevent changes in lipid profile (4). However, it has been observed that when more than 60% of the total energy is provided as MCT, gastrointestinal alterations such as abdominal distension or diarrhea occur. For this reason, experts suggest limiting its use to 30% of total energy (4).

In our study, we observed that MCT consumption varied between 4% and 27% of total energy. However, two cases had low consumption of MCT (<10% of total energy), attributable to the fact that this product is not subsidized by the State. Thus, families must cover the cost, which becomes a limitation for use, especially among low-income families. This is a very different scenario from the special ketogenic formula, which is subsidized by the State for all patients diagnosed with GLUT1-DS, a benefit that lasts for life. Every patient diagnosed with this pathology enters the complementary feeding program (PNAC in Spanish) of the Chilean Ministry of Health.

Some studies have observed that prescribing MCT between 12% and 20% of total energy reduces plasma levels of triglycerides and LDL cholesterol, reducing the risk of coronary heart disease (17). Our results show that the group with GLUT1-DS in KDT consumed 17% of the energy as MCT and that this did not cause significant differences in cholesterol, LDL, HDL and triglyceride values compared to the control group.

MUFA has been associated with various benefits for cardiovascular health. There is evidence to suggest that replacing carbohydrate consumption with MUFA can increase HDL cholesterol levels while decreasing both total cholesterol and LDL cholesterol (18).

In our cohort, it was determined that 22.4% of the total energy was from MUFA fatty acids and the HDL value was 60.7 ± 14.0 mg/dL, which is within the reference range. If we consider that the GLUT1-DS group receives on average 80% of its total energy as fats, it is important to highlight that 50% of this consists of MUFA+PUFA fats, indicating that KDT does not cause deleterious alterations in the lipid profile. Furthermore, an inverse correlation was observed between MUFA fat intake, triglycerides and VLDL levels.

Various benefits have been associated with the consumption of PUFA in the diet, especially those related to the omega-3 family. For example, DHA, which comprises 40% of the structures of the brain, 40% of the retina, and 50% of the weight of neurons, is an essential nutrient during childhood (12). In addition, it is a precursor to a series of prostaglandins-3 (inhibit platelet aggregation, reduce inflammation and hyperlipemia), a group of thromboxane-3 and leukotrienes-5 (all eicosanoids). It has been observed that the intake of omega-3 has a significant effect on reducing triglyceride and cholesterol levels, thus providing important benefits for cardiovascular health (12, 15).

In the biosynthesis process in peroxisomes, EPA (20:5n-3) and DHA (22:6n-3) can be synthesized by the human body from ALA (18:3n-3). However, the conversion rate is quite small and only a fraction of all the ALA consumed ends up being transformed into EPA and DHA. For this reason, direct consumption of both fatty acids through the diet is recommended (12). In our study, subjects of GLUT1-DS had a consumption of 25.8 ± 1.7 g/day of ALA, mainly from canola oil, which contains about 10% ALA and corresponds to 1.5% of total energy.

The recommended consumption of EPA + DHA varies according to age (Supplementary Table 1). An article discusses how vegetarian and vegan diets in children and adolescents can have a higher consumption of PUFA through vegetable oils; however, the contribution of DHA is frequently below the recommended intake (19). In our study, EPA + DHA was analyzed, and subjects achieved a contribution of 572.4 ± 880.7 mg/day from fish oil, which is under the recommendations in 50% of the subjects and could be compensated with ALA intake.

To achieve a balance between the synthesis of omega 6 and omega 3 fatty acids, because they share the same enzymes to form docosapentaenoic acid (22:5 omega-6) and DHA, a ratio of 5:1 has been established between both families (n-6: n-3), and some have suggested up to 7.5:1. Recently, the use of this ratio has been debated and its clinical value has been questioned. Therefore, the use of the relationship between arachidonic acid and EPA, and the omega-3 index has emerged, which appears to be a more robust biomarker in clinical and human studies (15). In the GLUT1-DS group, the ratio was 4.5:1. However, it should be emphasized that the reason could not be established in the control group, because the record intake of types of fat was not recorded separately, but as a total.

The GLUT1-DS group had an average vitamin C intake of 390 mg/day, mainly from supplementation, evidencing a contribution above the recommendations established by RDI. It is important to note that our cohort receives this supplementation to prevent deficiencies due to dietary restrictions of fruits, but also because this vitamin directly participates in preventing lipoperoxidation of cell membranes (4, 20). It is relevant to mention that the impact of vitamin C supplementation on the lipid profile in young and adult subjects has been investigated, but conclusive evidence has not been established in this regard. Only lower levels of triglycerides and LDL cholesterol have been observed in individuals with hypercholesterolemia (21).

Regarding vitamin D intake, the GLUT1-DS group received an average supplementation of 660 IU/day to complete the recommended requirements according to age and gender. Although there are no studies that evaluate the effect of supplementation in subjects similar to those in our study, evidence suggests that deficiency of this vitamin may be related to a higher risk of dyslipidemia, and correcting it could be beneficial for individual health, especially among adults (22, 23).

Regarding biochemical parameters, some articles have reported alterations in the lipid profile in the first months after starting KDT; however, after treatment continues, levels tend to normalize in 60% of cases. Different strategies are reported in the literature to prevent hyperlipidemia, such as increasing the consumption of MCT, olive oil, omega-3, or carnitine supplementation. Experts also recommend reducing the consumption of cholesterol, animal fat, and coconut oil, among others (24–26).

Concerning correlations, the direct relationship between saturated fat and the level of ALT observed in this study is relevant, since ALT levels have been considered as a screening indicator for non-alcoholic fatty liver in children who are overweight (27). We did not observe ea large number of subjects with altered ALT levels in our sample, despite having 50% of the subjects being obese or overweight and a high intake of saturated fat, mainly from MCT oil. This finding suggests that saturated fats from MCT oil may trigger a metabolic response distinct from established scientific evidence and potentially have no impact on liver enzymes. To validate this finding, additional studies are necessary.

Although this study provides valuable information, it has limitations. These limitations were a small sample size and observational study design. In addition, the use of 24-h recall as a tool to estimate intake of such specific nutrients may generate inaccurate results and, finally, the lack of nutritional information on the omega-3 and omega-6 intake of foods limited our analysis. However, it highlights the importance of a carefully planned diet in the management of GLUT1-DS and suggests areas for future research, such as the long-term impact of KDT on the cardiovascular health of patients.

## 5 Conclusion

The ketogenic diet plays a critical role in managing GLUT1-DS, a neurological disease caused by mutations in the SLC2A1 gene. This diet is effective in controlling seizures and improving cognitive development in patients.

The results showed that patients’ diets were mainly composed of fats, with a higher consumption of saturated fats, MUFAs, and PUFAs among GLUT1-DS subjects compared to the control group. The use of MCT, an integral part of KDT, was highlighted. Despite the high consumption of saturated fats, no significant differences were observed in the lipid profile or liver function of GLUT1-DS patients compared to controls. Thus, MUFAs and PUFAs, especially EPA+DHA, should be included in KDT to prevent changes in the lipid and liver profile and to avoid adverse effects that could later cause pathologies associated with malnutrition.

We recommend that KDT should always consider the use of MCT and provide 50% of total fats as MUFA+PUFA, especially EPA and DHA. We also suggest continuous surveillance, monitoring in the clinic, and long-term biochemistry.

## Data availability statement

The original contributions presented in this study are included in this article/Supplementary material, further inquiries can be directed to the corresponding author.

## Ethics statement

The studies involving humans were approved by the Comité de ética del Instituto de Nutrición y Tecnología de los Alimentos de la Universidad de Chile. The studies were conducted in accordance with the local legislation and institutional requirements. Written informed consent for participation in this study was provided by the participants’ legal guardians/next of kin.

## Author contributions

MS: Conceptualization, Data curation, Methodology, Writing – original draft, Writing – review and editing. ML-W: Conceptualization, Formal analysis, Methodology, Writing – review and editing, Project administration. VP: Data curation, Investigation, Writing – review and editing. CA: Writing – review and editing. VC: Supervision, Conceptualization, Writing – review and editing, Funding acquisition.
