# Supplementary material for: Analysis of dietary fats intake and lipid profile in Chilean patients with glucose transport type 1 deficiency syndrome: similarities and differences with the reviewed literature
Source: Front Nutr. 2024 May 16;11:1390799. doi: 10.3389/fnut.2024.1390799 (PMC11137239; doi:10.3389/fnut.2024.1390799)
Supplement: Supplementary file 1 [file Table_1.docx]

Supplementary Material

# Supplementary Figures and Tables

| **Supplementary Table:** Recommendations for both groups (Control and GLUT-DS) for different types of fat, vitamin C, and D according to age group. | | |
| --- | --- | --- |
| Nutrients | Age group | |
|  | 3- 18 years | > 18 years |
| Total fat (%E) | 25-35% | 20-35% |
| SFA (%E) | ≤8 % | ≤ 10% |
| MUFA (%E) | is calculated by difference with respect to the other fats | |
| PUFA (%E) | 11% | 6-11% |
| Omega 3 (%E)  : | Omega 3 total: NA  ALA: 0.6 - 1.2% | Omega 3 total: 0.5-2%  ALA: > 0.5% |
|  | EPA+ DHA (mg/day)  2-4 years: 100-150  4-6 years: 150-200  6-10 years: 200-250 | EPA+ DHA (mg/day)  2500 - 9000 |
| Omega 6 (%E) | α-linolenic acid: 5-10 | 2.5-9 |
| Cholesterol (mg/day) | NA | < 350 |
| Vitamin D (IU/day) | 600 | 600 |
| Vitamin C (mg/day) | 1-3 years: 15  4-8 years:25  9-13 years: 45  14-18 years (women): 65  14-18 years (men): 75 mg | Men: 75  Woman: 90 |
| Recommendation according to age group (12,13,14¨) NA: not available; %E: percentage of total energy; SFA: saturated fat; MUFA, monounsaturated fat: PUFA: polyunsaturated fat; ALA: α-linolenic acid; EPA: eicosapentaenoic acid; DHA: docosahexaenoic acid; IU: international unit. | | |
